# A national cross-sectional survey of health literacy of caregivers attending Canadian pediatric emergency departments

**DOI:** 10.1371/journal.pone.0314826

**Published:** 2024-12-20

**Authors:** Manasi Rajagopal, Samina Ali, Keon Ma, Maryna Yaskina, Andrea Morrison, Kurt Schreiner, Julie Leung, Shannon Scott, Darcy Beer, Paul Clerc, Tyrus Crawford, Serge Gouin, Naveen Poonai, Tania Principi, Antonia Stang, Laura Weingarten, Janet Curran, on behalf of the Pediatric Emergency Research Canada Family Needs Study Team

**Affiliations:** 1 Faculty of Medicine & Dentistry, Department of Pediatrics, University of Alberta, Edmonton, Alberta, Canada; 2 Women & Children’s Health Research Institute, University of Alberta, Edmonton, Alberta, Canada; 3 Department of Pediatrics, Cumming School of Medicine, University of Calgary, Calgary, Alberta, Canada; 4 CommunicateMD, Grafton, Wisconsin, United States of America; 5 Department of Pediatrics, PEAK Research Team, University of Alberta, Edmonton, Alberta, Canada; 6 Pediatric Parent Advisory Group, ECHO Research Program, University of Alberta, Edmonton, Alberta, Canada; 7 Faculty of Nursing, University of Alberta, Edmonton, Alberta, Canada; 8 Children’s Hospital Research Institute of Manitoba, University of Manitoba, Winnipeg, Manitoba, Canada; 9 Department of Pediatrics, Division of Emergency Medicine, University of British Columbia and BC Children’s Hospital Research Institute, The Pediatric Research in Emergency Therapeutics (PRETx) Program, Vancouver, British Columbia, Canada; 10 Department of Pediatrics, University of Ottawa, Ottawa, Ontario, Canada; 11 CHU Ste Justine, Montreal, Quebec, Canada; 12 Departments of Paediatrics, Internal Medicine, Epidemiology & Biostatistics, Schulich School of Medicine & Dentistry, Western University, London, Ontario, Canada; 13 Department of Pediatrics, McMaster University, Hamilton, Ontario, Canada; 14 Faculty of Health Professions, School of Nursing, Dalhousie University, Halifax, Nova Scotia, Canada; Yarmouk University, JORDAN

## Abstract

**Background:**

Health literacy assessment is key to better meeting family needs and developing informed strategies to promote positive health outcomes for children. The objective of this study was to describe the health literacy of caregivers who use Canadian pediatric emergency departments and relate it to demographic and visit-specific variables.

**Methods:**

This study utilized a descriptive, cross-sectional survey design with medical record review. A bilingual survey was electronically administered to caregivers presenting to 10/15 Canadian pediatric emergency departments. Health literacy was assessed using the Newest Vital Sign tool.

**Results:**

1957 caregivers completed the Newest Vital Sign assessment. Caregivers’ mean age was 37.8 ± 7.7 years, 74.3% (1449/1950) were mothers and 51.9% (993/1912) had a university/professional degree. 12.0% (235/1957) had a high likelihood of limited health literacy, 16.5% (323/1957) had possible limited health literacy and 71.5% (1399/1957) demonstrated adequate health literacy. Adequate health literacy scores were associated with having a university/professional degree [aOR 1.47 (95% CI 1.11–1.94)] and having a household annual income of over $25,000 [aOR 4.10 (2.66–6.31)]; they were inversely associated with having a total of 4 or more children [aOR 0.61 (0.40–0.91)] and having a main language at home other than English or French [aOR 0.32 (0.23–0.43)].

**Interpretation:**

With over 1/4 caregivers facing health literacy challenges, health care providers in emergency departments must be cognizant of their communication and education approach when caring for families and providing at-home care guidance. Clinicians should consider applying health literacy principles to all family encounters to help address healthcare disparities.

## Introduction

Health literacy refers to the collective skillset that is required for an individual to make optimal health care decisions for themselves or their child [1,2]. It encompasses one’s capacity to obtain, interpret and apply basic health-related information [3,4]. Health literacy considers prior knowledge, information-seeking, oral and written communication, numeric ability, and the ability to advocate for oneself in health care settings [5]. Health literacy is a stronger predictor of health status than socioeconomic status, age, or ethnic background [6].

Limited health literacy is associated with fewer preventative and primary care visits, increased hospitalizations [7], worse disease-specific outcomes [5], and higher mortality rates [8]. Previous literature has shown that caregivers with low health literacy have decreased knowledge about medication dosing for their children, and make more errors interpreting instructions and warnings [9,10]. Caregivers with lower health literacy also overestimate illness severity and overutilize healthcare resources, including greater presentations for non-urgent reasons to emergency care services [5,11–13]. Pediatric emergency departments (PEDs) are inherently high-stress, noisy, and unpredictable environments for most families, making it challenging for caregivers to follow and remember healthcare instructions [14,15]. This is compounded by limited health literacy for many of these caregivers. Evaluating health literacy and incorporating appropriate strategies to enhance information comprehension and retention is crucial to providing high-quality family-centered care [12].

Health literacy is dynamic, impacted by the evolving relationship between the person and the health care system they are navigating [5,12]. Currently, there is limited knowledge of caregivers’ health literacy levels in PEDs. A 2008 federal report suggests that up to 60% of adult Canadians have low health literacy [16]. More specifically, with regards to caregivers of children, a previous systematic review has estimated that approximately 30% of American caregivers presenting to PEDs have low health literacy [17]. To our knowledge, no analogous data exists in Canada, which our patient partners felt was critical to explore. The objective of this study was to describe the health literacy of a population of caregivers who attended Canadian PEDs, as well as the association between caregiver health literacy and socio-demographic and ED visit characteristics.

## Methods

### Design and setting

This is a sub-study of data collected as part of a larger descriptive, cross-sectional survey study, which sought to identify the needs of families presenting to Canadian PEDs. The survey was created with patient partners, utilizing item generation and reduction, along with pre-, pilot, and sensibility testing to ensure face and content validity. A convenience sample of caregivers was enrolled from 10 academic tertiary care PEDs, all of whom are part of Pediatric Emergency Research Canada (https://perc-canada.ca/): 1. BC Children’s Hospital (Vancouver, British Columbia), 2. Stollery Children’s Hospital (Lead Site; Edmonton, Alberta), 3. Alberta Children’s Hospital (Calgary, Alberta), 4. Winnipeg Children’s Hospital (Winnipeg, Manitoba), 5. Children’s Hospital at London Health Sciences Center (London, Ontario), 6. Hospital for Sick Children (Toronto, Ontario), 7. McMaster Children’s Hospital (Hamilton, Ontario), 8. Children’s Hospital of Eastern Ontario (Ottawa, Ontario), 9. CHU Ste. Justine (Montreal, Quebec), and 10. IWK Health Centre (Halifax, Nova Scotia). Each site completed one week of recruitment per season, for a total of 4 weeks over a one-year period. Seasons were defined as Fall (September 1 –November 30), Winter (December 1 –February 28), Spring (March 1 –May 31) and Summer (June 1 –August 31). Due to variations in institutional approval times, study start dates were staggered across sites. Participants were enrolled between October 2018 and March 2020. Recruiting hours varied based on staff availability and resources, but generally ranged from 7am-11pm. A sample of approximately 2000 caregivers presenting to the pediatric ED were targeted to be enrolled in the study based on a goal of 200 caregivers recruited per site. We limited recruitment at each site to a maximum of 50 patients per week of recruitment, to avoid over-representation of any one site. Formal sample size calculations were not performed, as this was a nested secondary analysis within a larger study of global family needs in the PED [18,19]. Two caregivers with lived experience, who were not study participants, were co-investigators and contributed to the study from its inception including study design, results interpretation, and knowledge translation plan. The site leads and research coordinators had access to information that could identify individual participants at their own site, only, during data collection. All data were anonymized prior to analyses and sharing of results within the research team.

### Study population

All consenting caregivers with children aged 0–17 years who presented to a participating PED with any chief complaint were potentially eligible. Caregivers were excluded if (a) unable to read and write English or French; (b) their child remained medically unstable throughout their ED stay; (c) there was a suspicion of child abuse; (d) their child presented with an altered level of consciousness; or (e) they were not the child’s legal guardian. Individuals were only permitted to participate once in the study.

### Data collection

Screening and enrollment were completed by trained research personnel. The lead research coordinator at each site was trained by the principal investigator and national study coordinator. Written informed consent was obtained prior to data collection. Caregivers independently completed an electronic questionnaire on a research study tablet during their ED visit, containing questions regarding demographics, reason for their visit, state anxiety via the State Trait Anxiety Inventory-State (STAI-S) [20], and health literacy via the Newest Vital Sign (NVS) [21,22]. The novel survey which we employed underwent item generation and reduction (6 member expert panel), as well as pre-testing (8 participants contacted via email), pilot testing (10 participants in ED setting), and sensibility testing (10 participants in ED setting). This allowed for assessment of face and content validity prior to implementation; criterion validity and internal consistency were not assessed. Caregivers were able to skip questions any questions that they wished and return back to modify earlier questions. Those who did not answer any of the NVS questions were assumed to have not attempted it and were excluded from analysis. Missed responses were not permuted. The entire survey, including the NVS, was administered digitally via a tablet and was completed independently by the caregiver participant. The research assistant did not assist the participants with filling out any part of the NVS. The survey was administered using the Research Electronic Data Capture (REDCap) platform (University of Alberta) [23]. Additional variables (e.g., ED length of stay (i.e., length of full visit from triage to discharge/admission, LOS), acuity score, discharge disposition) were extracted through medical record review and entered directly into the database. Data entry into REDCap utilized a secure web-based interface, and only authorized research personnel were granted user access to the database.

### Outcomes

The primary outcome measure was caregiver health literacy as assessed by the NVS, which consists of six health-related questions that require interpretation of a nutrition label [21,22]. The NVS was selected as it is one of the most sensitive and discriminatory tools to detect limited health literacy in caregivers of young children [24–26]. Its ease of administration (2–6 minutes) is ideal for the ED setting [27], which was corroborated by our patient partners, and its availability in English and French makes it suitable for Canadian hospitals [21]. This study utilized the Mansfield, et al. Canadian adaptation of the NVS, which utilizes multiple choice questions and is intended for self-administration [21]. This adaptation uses modified nutrition labelling to comply with Canada’s Food and Drug Regulations guidelines [21]. With one point per correct answer, NVS scores may range from zero to six. Original scoring guidelines categorize the scores as follows: high likelihood of limited literacy (0–1), possibility of limited literacy (2–3), and adequate literacy (4–6) [22]. These original scoring guidelines were established using data from an older primary care adult population. Based on more recent research conducted within younger parent populations, the following modified scoring guidelines have also been suggested: low literacy (0–4), and adequate literacy (5–6) [24]. Both scoring guidelines have been used to report data within this paper, given that parents of pediatric patients tend to be younger.

Secondary outcome measures included caregiver socio-demographic characteristics (e.g., age, sex, annual household income), child demographics (e.g., age, sex), ED visit details (Canadian Triage and Acuity Scale score [28], ED LOS), past medical history, and caregiver anxiety. Caregiver state anxiety was measured with the STAI-S, Form Y, a validated and commonly used scale that is available in French and English [20,29]. The STAI-S Form Y consists of 20 questions to assess the current state of anxiety that an individual is experiencing in the moment. It includes questions such as: “I am tense; I am worried” and “I feel calm; I feel secure,” and these questions are scored such that the scoring range for the tool is 20–80 [30]. A higher score indicated greater caregiver anxiety, while a lower end score indicates the opposite [31,32].

### Statistical analysis

Statistical analysis was performed using SAS Ver. 9.4 (SAS Institute Inc., Cary, NC, USA). Descriptive statistics (means, medians, standard deviations, ranges) were used to summarize continuous variables (e.g., age, ED LOS) while frequency distributions were used to summarize categorical variables (e.g., sex, health literacy). Differences between groups (e.g., provinces) have descriptive comparisons reported. Multivariable logistic regression was used to ascertain effects of *a priori* selected variables (e.g., age, sex, education, income, province, previous number of ED visits) on caregiver health literacy. For the regression analyses, health literacy was further categorized into a binary variable (limited vs adequate). Both the original and revised NVS scoring thresholds are used to report data within this paper. Results from the regression models were reported using odds ratios and 95% confidence intervals, and a p-value less than 0.05 was considered statistically significant.

### Ethics approval

This study was approved by the Health Research Ethics Board at University of Alberta (Pro00075437), and by individual research ethics boards at each site.

## Results

A total of 2005 caregivers consented to participate; 1957/2005 (97.6%) completed the NVS health literacy assessment.

### Demographic characteristics

Mean (SD) caregiver age was 37.8 (7.7) years and 74.7% (1457/1950) were female. Most (72.6%; 1417/1953) were English speaking, and 51.9% (993/1912) had a completed university/professional degree. Caregivers reported a mean (SD) state anxiety score of 37.9 (11.1) (Table 1).

**Table 1:** Caregiver demographics.

Variable	n (%)
**Parent/Caregiver Age (years**), mean (SD) (n = 1894)	37.8 (7.7)
**Relationship to Child**, (n = 1950) Mother Father Grandparent Other	1449 (74.3) 470 (24.1) 13 (0.7) 18 (0.9)
**Total number of children,** (n = 1888) 1 2 3 4 >4	450 (23.8) 848 (44.9) 371 (19.7) 150 (7.9) 69 (3.7)
**Main language spoken at home**, (n = 1953) English French Spanish Mandarin Chinese/ Cantonese Hindi/ Urdu/ Punjabi Arabic Other	1417 (72.6) 206 (10.5) 43 (2.2) 46 (2.3) 41 (2.1) 40 (2.0) 160 (8.2)
**Highest Level of Education**, (n = 1912) Elementary School High School/ some High School Diploma/Certificate Some University University/Professional Degree	7 (0.4) 183 (9.6) 402 (21.0) 327 (17.1) 993 (51.9)
**Annual Household Income,** (n = 1699) Less than or equal to $25,000 $25,001 - $50,000 $50,001 - $75,000 $75,001 - $100,000 Greater than $100,000	134 (7.9) 266 (15.7) 236 (13.9) 297 (17.5) 766 (45.1)
**Province of Residence,** (n = 1957) British Columbia Alberta Manitoba Ontario Quebec Nova Scotia	199 (10.2) 431 (22.0) 115 (5.9) 882 (45.1) 208 (10.6) 122 (6.2)
**Caregiver Anxiety (STAI) Score during ED visit,** mean (SD) (n = 1813)	37.9 (11.1)

Accompanying children who were seen at the ED were 48.1% (940/1956) female with a mean (SD) age of 5.9 (5.0) years; 32.2% (628/1952) had one or more previous hospitalizations, and 81.5% (1587/1948) had previously ED visits. The median (IQR) ED LOS was 3.9 (2.6, 6.1) hours (Table 2).

**Table 2:** Child and ED visit demographics.

Variable	n (%)
**Child Age (years),** mean (SD) (n = 1955)	5.9 (5.0)
**Child Sex (female),** (n = 1956)	940 (48.1)
**CTAS Category,** (n = 1943) 1. Resuscitation 2. Emergency 3. Urgent 4. Semi-urgent 5. Non-urgent	9 (0.5) 359 (18.5) 995 (51.2) 506 (26.0) 74 (3.8)
**Triage Time,** (n = 1950) Day (08:01–16:00) Evening (16:01–00:00) Overnight (00:01–08:00)	1339 (68.7) 527 (27.0) 84 (4.3)
**Discharge Disposition,** (n = 1955) Discharged Admitted Transferred to another facility Other	1669 (85.4) 251 (12.8) 9 (0.5) 26 (1.3)
**ED Length of Stay (hours),** median (IQR) (n = 1932)	3.9 (2.6,6.1)
**Number of Previous ED Visits,** (n = 1948) 0 1–5 6–10 >10	361 (18.5) 1143 (58.7) 270 (13.9) 174 (8.9)
**Number of Previous Hospitalizations,** (n = 1952) 0 1–5 6–10 >10	1324 (67.8) 546 (28.0) 26 (1.3) 56 (2.9)
**Chronic Disease,** (n = 1949)	367 (18.8)

### Health literacy

Based on the original NVS scoring system, twelve percent (235/1957) of caregivers had a high likelihood of limited health literacy, 16.5% (323/1957) had possible limited health literacy and the remaining 71.5% (1393/1957) demonstrated adequate health literacy. When analyzed using revised scoring guidelines suggested by Morrison et al [25], the proportion of caregivers with limited health literacy (including ‘high likelihood’ and ‘possible’ limited literacy) rose to 43.7% (885/1957). The median (IQR) NVS raw score across all participants was 5 (3,6) out of 6. (Table 3) Caregiver health literacy, by province, is presented in Table 4.

**Table 3:** Health literacy of caregivers presenting to the pediatric ED, as measured by the Newest Vital Sign (NVS) (n = 1957).

	All Caregivers
**NVS Raw Score** (maximum possible score = 6) Mean (SD) Median (IQR)	4.3 (1.9) 5 (3,6)
**NVS Health Literacy Category (original)**^**a**^ 0–1: High likelihood of limited literacy, n (%) 2–3: Possibility of limited literacy, n (%) 4–6: Adequate literacy, n (%)	235 (12.0) 323 (16.5) 1399 (71.5)
**NVS Health Literacy Category (modified**)^**b**^ 0–4: Low literacy, n (%) 5–6: Adequate literacy, n (%)	885 (43.7) 1102 (56.3)
**NVS Accuracy by Question** n(%) Question 1 Correct (n = 1953) Question 2 Correct (n = 1948) Question 3 Correct (n = 1941) Question 4 Correct (n = 1943) Question 5 Correct (n = 1947) Question 6 Correct (n = 1462)	1380 (70.7) 1558 (80.0) 1278 (65.8) 1320 (67.9) 1465 (75.2) 1374 (94.0)

^a^ based on original scoring guidelines by Weiss et al. 2005 [22].

^b^ based on modified scoring guidelines by Morrison et al. 2014 [12].

**Table 4:** Health literacy (Newest Vital Sign score) of caregivers presenting to the pediatric ED, by province (n = 1957).

	British Columbia *(n = 199)*	Alberta *(n = 431)*	Manitoba *(n = 115)*	Ontario *(n = 882)*	Quebec *(n = 208)*	Nova Scotia *(n = 122)*	All Caregivers *(n = 1957)*
**NVS Raw Score** Mean (SD) Median (IQR)	4.6 (1.7) 5.0 (4.0,6.0)	4.4 (1.9) 5.0 (3.0,6.0)	3.7 (2.2) 4.0 (2.0,6.0)	4.2 (1.8) 5.0 (3.0,6.0)	4.2 (1.9) 5.0 (3.0,6.0)	4.5 (1.7) 5.0 (4.0,6.0)	4.3 (1.9) 5.0 (3.0,6.0)
**NVS Health Literacy Category (original**)^**a**^ n (%) 0–1: High likelihood of limited literacy 2–3: Possibility of limited literacy 4–6: Adequate literacy	17 (8.5) 23 (11.6) 159 (79.9)	49 (11.4) 71 (16.5) 311 (***72***.2)	23 (20.0) 24 (20.9) 68 (59.1)	107 (12.1) 147 (16.7) 628 (71.2)	28 (13.5) 39 (18.8) 141 (67.8)	11 (9.0) 19 (15.6) 92 (75.4)	235 (12.0) 323 (16.5) 1399 (71.5)
**NVS Health Literacy Category, (modified)**^**b**^ n (%) 0–4: Low literacy 5–6: Adequate literacy	72 (36.2) 127 (63.8)	178 (41.3) 253 (58.7)	67 (58.3) 48 (41.7)	398 (45.1) 484 (54.9)	96 (46.2) 112 (53.8)	44 (36.1) 78 (63.9)	855 (43.7) 1102 (56.3)

^a^ based on original scoring guidelines by Weiss et al. 2005 [22].

^b^ based on modified scoring guidelines by Morrison et al. 2014 [12].

### Factors associated with health literacy

Relevant univariate modeling is presented in S1 and S2 Tables. In multivariable logistic regression modeling, the following factors were associated with adequate health literacy in caregivers, as defined by an NVS score of 4–6 (original scoring guidelines): having a university/ professional degree (vs diploma/certificate or some university) [OR 1.47 (95% CI (1.11, 1.94)], and having a household income of $25,001 - $100,000 [OR 4.10 (2.66, 6.31)] or greater than $100,000 (OR 9.80 (6.09, 15.77)] (Table 5).

**Table 5:** Adjusted odds ratios for likelihood of having adequate health literacy.

**Likelihood of having adequate health literacy, as defined by an NVS score of 4–6** ^**a**^		
**Variable**	**Odds Ratio (95% CI)**	**p-value**
Total number of children (4+ vs 1)	0.61 (0.40–0.91)	0.02
Main language spoken at home (Language other than English/French vs English)	0.32 (0.23–0.43)	<0.0001
Education (University/Professional degree vs Diploma/Certificate or Some university)	1.47 (1.11–1.94)	0.007
Household income ($25,001 - $1000,000 vs $25,000 and under) (> $100,000 vs $25,000 and under)	4.10 (2.66–6.31) 9.80 (6.09–15.77)	<0.0001 <0.0001
**Likelihood of having adequate health literacy, as defined by an NVS score of 5-6** ^**b**^		
**Variable**	**Odds Ratio (95% CI)**	**p-value**
Main language spoken at home (Language other than English/French vs English)	0.38 (0.28–0.52)	<0.0001
Education (University/Professional degree vs Diploma/Certificate or Some university)	1.55 (1.22–1.96)	0.0003
Household income ($25,001 - $1000,000 vs $25,000 and under) (> $100,000 vs $25,000 and under)	3.56 (2.22–5.71) 7.14 (4.36–11.69)	<0.0001 <0.0001

^a^ based on original scoring guidelines by Weiss et al. 2005 [22]; ^b^ based on modified scoring guidelines by Morrison et al. 2014 [12].

Factors associated with caregivers not having adequate health literacy included having a total of 4 or more children [OR 0.61 (0.40, 0.91)], and the main language at home being a language other than English or French [OR 0.32 (0.23, 0.43)]. Caregiver’s age, province, relationship to child, and reason for ED visit were not associated with the NVS score. Education, household income and province were checked for collinearity, which was not found.

## Discussion

Among our cohort of almost 2000 caregivers from ten Canadian PEDs, over one-quarter faced health literacy challenges. Limited health literacy was associated with lower educational attainment, lower household income, having 4 or more children and having a main language at home other than English or French. This information can help healthcare providers (HCPs) understand the communication needs and challenges of the families they serve and allow them to adapt their information-sharing in a manner that supports wider understanding.

While there are no comparable Canadian studies available to refer to, our results are congruent with American data where low health literacy was reported in approximately one-third of caregivers presenting to PEDs and over one-quarter of American parents in general [33]. In contrast, the prevalence of low health literacy in adult EDs is reported to be higher at 40% [34]. This is likely because older age is associated with increased health literacy challenges and parents attending PEDs, such as in our study, are generally younger in age, than the overall adult population [17]. Additionally, limited health literacy is more prevalent in non-parents than in parents, and all participants in our study were caregivers to children, by virtue of our inclusion criteria [33]. When utilizing the revised scoring guidelines for health literacy [24], the proportion of caregivers in our study with limited health literacy rose even higher, to 43.7%. This prevalence estimate is still lower than the 55% reported in an analogous American study; notably their study population had educational attainment of college degree in only 30% of their population, compared to >50% in our study sample. [24].

Our study suggests that a notable proportion of caregivers, between 29–44%, may have difficulty understanding or interpreting important health information. This has significant implications for children’s health and safety. Health literacy is not routinely assessed in the ED setting, and providers typically lack a pre-existing care relationship with their patients. Health care providers should consider implementing a ‘universal precautions’ approach to help mitigate negative outcomes associated with low health literacy, such as medication errors, increased ED use, worse health outcomes, and increased health care costs [35,36]. A universal precautions approach assumes that all caregivers may have some difficulty understanding and applying health information. Interventions are then aimed at enhancing communication and teaching with *all* caregivers, by utilizing strategies such as: teach-back and show-me methods, using visual aids for education, developing easy-to-read educational materials, and encouraging questions [35,36]. It is also recommended to have health information available in various formats (e.g., print-based, visual, video, website) and in multiple languages [35,37]. While it has been previously reported that many caregivers prefer information in a web-based format, those with lower health literacy are less likely to use the internet and often prefer paper-based information that is provided directly by an HCP, to ensure source credibility [37].

Our study demonstrates that limited health literacy is associated with low educational attainment and low annual household income, which is consistent with prior research [11,24,38]. A previous PED study found the prevalence of limited health literacy to be significantly lower in caregivers with a completed university degree (29%) than in those who had completed high school only (72%) [24]. Our study also demonstrates an increased odds of low health literacy in caregivers who have four or more children. Previous research on this has been inconclusive, with one study of low-income mothers reporting that those with more children had lower health literacy [39], while another study did not identify an association [40].

Our study demonstrates an increased odds of limited health literacy scores in caregivers whose primary language at home was not English or French. In our study cohort, almost 1/5 caregivers had a primary language other than English or French. Caregivers with language barriers or those communicating in their non-dominant language may need specialized interventions to ensure that key health information is not missed. Strategies to address language differences include asking about language preference at registration, using language assistance services, having written materials available in multiple languages and visual formats, and using plain non-medical language [35].

Caregivers in our study reported a mean STAI-S, Form Y anxiety score of 38, which is comparable to that previously reported in other Canadian PED studies (range: 32 to 42) [41,42]. In a striking comparison, state STAI scores reported for international soldiers ranges from 32–39 [43,44]. Interestingly, our univariable analyses did not demonstrate a relationship between anxiety and health literacy. However, previous work from our team has demonstrated that higher anxiety (as measured by the STAI-S) is associated with caregivers reporting less comfort caring for their child at home, after ED discharge [18]. We need to improve how we define and measure stress and health literacy in the ED setting. Further, the relationship between emergency visits, anxiety, and health literacy testing requires additional exploration through both quantitative and qualitative analyses.

## Conclusion

Over one in four caregivers presenting to Canadian PEDs have NVS scores that are associated with low health literacy. This may limit their ability to meaningfully participate in health-care decisions for their child. It is essential that HCPs understand their population’s health literacy so that they may develop informed communication strategies and interventions to best meet the family’s needs. Strategies that may be of benefit include adopting a ‘universal precautions’ approach of using health literacy principles with all caregivers, addressing language differences, using interactive teaching methods, and having easy-to-read educational materials available via multiple modalities. Future research to investigate care-seeking behaviours and information preferences among caregivers with lower health literacy may help inform more targeted health literacy interventions.

### Limitations

Due to logistical constraints, the study survey was only offered in English and French. Our patient partners have highlighted the need for a future larger-scale study inclusive of caregivers coming from diverse cultural and linguistic backgrounds. The NVS assessment was completed independently by the caregiver using a tablet rather than administered verbally. Previous studies evaluating self-administration of the NVS found that those with low literacy skills may fail to complete the assessment [21,45]; this was not a significant barrier for our study participants (98% NVS completion rate). Notably, due to logistic constraints during data collection, we were unable to record the number of families who refused to participate in the overall study. Survey distribution was limited to the hours of research staff availability, recruiting a convenience sample, and thus under-representing the overnight period. Selection bias is possible as caregivers with limited literacy may have refused study participation. As such, this study may not fully represent the diversity of all families visiting PEDs. Measurement and recall bias are also risks to all survey studies. Finally, given the compassionate exclusion of families where children were critically ill, we were not able to enroll caregivers with the sickest children. Notably, these represent a small minority of patients in the PED.

## Supporting information

S1 Table Univariable logistic regression model for likelihood of having adequate health literacy (as defined by an NVS score of 4–6). (DOCX)

S2 Table Univariable logistic regression model for likelihood of having adequate health literacy (as defined by an NVS score of 5–6). (DOCX)
