# Supplementary material for: A national cross-sectional survey of health literacy of caregivers attending Canadian pediatric emergency departments
Source: PLoS One. 2024 Dec 20;19(12):e0314826. doi: 10.1371/journal.pone.0314826 (PMC11661602; doi:10.1371/journal.pone.0314826)
Supplement: S1 Table — (DOCX) [file pone.0314826.s001.docx]

**S1 Table: Univariable logistic regression model for likelihood of having adequate health literacy (as defined by an NVS score of 4-6)**

| **Variable** | **Odds ratio (95% CI)** | ***p-value*** | ***AUC*** |
| --- | --- | --- | --- |
|  |  |  |  |
| **Previous visits to ED** |  | 0.45 | 0.52 |
| 1-5 vs None | 1.07 (0.84, 1.37) | 0.58 |  |
| 6 or more vs None | 0.88 (0.68, 1.14) | 0.34 |  |
| **Previous hospitalizations** |  | 0.40 | 0.51 |
| 1-5 vs None | 1.45 (0.83, 2.52) | 0.19 |  |
| 6 or more vs None | 1.00 (0.81, 1.25) | 0.97 |  |
| **Chronic illness** |  | **0.005** | **0.53** |
| Unsure vs No | 0.60 (0.43, 0.84) | 0.003 |  |
| Yes vs No | 1.12 (0.86, 1.45) | 0.40 |  |
| **CTAS (4 categories)** |  | 0.51 | 0.52 |
| 3 – Urgent vs 1+2 | 1.17 (0.90, 1.52) | 0.2338 |  |
| 4 - Semi urgent vs 1+2 | 1.24 (0.93, 1.67) | 0.1467 |  |
| 5 - Non urgent vs 1+2 | 1.24 (0.71, 2.16) | 0.4535 |  |
| **Total number of children** |  | **<0.0001** | **0.58** |
| 2 vs 1 | **1.31 (1.01, 1.71)** | **0.04** |  |
| 3 vs 1 | 0.81 (0.60, 1.09) | 0.16 |  |
| 4+ vs 1 | **0.54 (0.38, 0.76)** | **0.0004** |  |
| **Main language at home** |  | **<0.0001** | **0.59** |
| French vs English | 0.99 (0.70, 1.39) | 0.95 |  |
| Other vs English | **0.31 (0.24, 0.40)** | **<0.0001** |  |
| **Education** |  | **<0.0001** | **0.61** |
| Elementary or High school vs Diploma/Certificate or Some university | **0.58 (0.42, 0.80)** | **0.0009** |  |
| University/Professional degree vs Diploma/Certificate or Some university | **1.91 (1.54, 2.38)** | **<0.0001** |  |
| **Household income** |  | **<0.0001** | **0.70** |
| $25,001 - $1000,000 vs $25,000 and under | **4.57 (3.08, 6.79)** | **<0.0001** |  |
| Greater than $100,000 vs $25,000 and under | **15.46 (10.11, 23.63)** | **<0.0001** |  |
| **Province** |  | **0.004** | **0.55** |
| Alberta vs Ontario | 1.05 (0.81, 1.35) | 0.72 |  |
| British Columbia vs Ontario | **1.61 (1.10, 2.34)** | **0.01** |  |
| Manitoba vs Ontario | **0.59 (0.39, 0.87)** | **0.009** |  |
| Nova Scotia vs Ontario | 1.24 (0.80, 1.92) | 0.33 |  |
| Quebec vs Ontario | 0.85 (0.62, 1.18) | 0.33 |  |
| **Relationship to child** |  | **0.0002** | **0.52** |
| Mother vs Father | 0.90 (0.71, 1.14) | 0.37 |  |
| Other vs Father | **0.20 (0.09, 0.42)** | **<0.0001** |  |
| **Caregiver’s age** | 1.**02 (1.002, 1.03)** | **0.03** | **0.53** |
| **STAI** | 0.998 (0.989, 1.008) | 0.75 | 0.50 |
