# Supplementary material for: A national cross-sectional survey of health literacy of caregivers attending Canadian pediatric emergency departments
Source: PLoS One. 2024 Dec 20;19(12):e0314826. doi: 10.1371/journal.pone.0314826 (PMC11661602; doi:10.1371/journal.pone.0314826)
Supplement: S2 Table — (DOCX) [file pone.0314826.s002.docx]

**S2 Table: Univariable logistic regression model for likelihood of having adequate health literacy (as defined by an NVS score of 5-6)**

| **Variable** | **Odds ratio (95% CI)** | **p-value** | **AUC** |
| --- | --- | --- | --- |
|  |  |  |  |
| **Previous visits to ED** |  | 0.54 | 0.51 |
| 1-5 vs None | 1.14 (0.90, 1.45) | 0.27 |  |
| 6 or more vs None | 1.09 (0.82, 1.44) | 0.55 |  |
| **Previous hospitalizations** |  | **0.07** | **0.52** |
| 1-5 vs None | 0.89 (0.73, 1.09) | 0.25 |  |
| 6 or more vs None | **1.56 (0.97, 2.51)** | **0.06** |  |
| **Chronic illness** |  | **0.14** | **0.52** |
| Unsure vs No | **0.74 (0.53, 1.03)** | **0.08** |  |
| Yes vs No | 1.08 (0.86, 1.36) | 0.51 |  |
| **CTAS (4 categories)** |  | 0.42 | 0.52 |
| 3 – Urgent vs 1+2 | 1.03 (0.81, 1.31) | 0.81 |  |
| 4 - Semi urgent vs 1+2 | 1.14 (0.87, 1.50) | 0.33 |  |
| 5 - Non urgent vs 1+2 | 1.44 (0.86, 2.41) | 0.17 |  |
| **Total number of children** |  | **<0.0001** |  |
| 2 vs 1 | **1.31 (1.04, 1.65)** | **0.02** |  |
| 3 vs 1 | 0.90 (0.68, 1.19) | 0.45 |  |
| 4+ vs 1 | **0.65 (0.47, 0.90)** | **0.009** |  |
| **Main language at home** |  | **<0.0001** | **0.56** |
| French vs English | 0.94 (0.70, 1.27) | 0.69 |  |
| Other vs English | **0.35 (0.27, 0.45)** | **<0.0001** |  |
| French vs Other | **2.68 (1.87, 3.83)** | **<0.0001** |  |
| **Education** |  | **<0.0001** | **0.61** |
| Elementary or High school vs Diploma/Certificate or Some university | **0.53 (0.38, 0.74)** | **0.0002** |  |
| University/Professional degree vs Diploma/Certificate or Some university | **1.88 (1.54, 2.28)** | **<0.0001** |  |
| **Household income** |  | **<0.0001** | **0.67** |
| $25,001 - $1000,000 vs $25,000 and under | **4.35 (2.76, 6.86)** | **<0.0001** |  |
| Greater than $100,000 vs $25,000 and under | **12.17 (7.66, 19.35)** | **<0.0001** |  |
| **Province** |  | **0.002** | **0.55** |
| Alberta vs Ontario | 1.17 (0.93, 1.48) | 0.19 |  |
| British Columbia vs Ontario | **1.45 (1.06, 1.99)** | **0.02** |  |
| Manitoba vs Ontario | **0.59 (0.40, 0.87)** | **0.008** |  |
| Nova Scotia vs Ontario | 1.46 (0.98, 2.16) | 0.06 |  |
| Quebec vs Ontario | 0.96 (0.71, 1.30) | 0.79 |  |
| **Relationship to child** |  | **0.0003** | **0.53** |
| Mother vs Father | 0.84 (0.68, 1.04) | 0.11 |  |
| Other vs Father | **0.16 (0.06, 0.40)** | **<0.0001** |  |
| **Caregiver’s age** | **1.02 (1.004, 1.03)** | **0.01** | **0.54** |
| **STAI** | 0.998 (0.990, 1.006) | 0.63 | 0.50 |
